# Duration of delayed diagnosis in HIV/AIDS patients in Iran: a CD4 depletion model analysis

**DOI:** 10.3389/fpubh.2023.1029608

**Published:** 2023-04-17

**Authors:** Mehdi Sharafi, Alireza Mirahmadizadeh, Jafar Hassanzadeh, Mozhgan Seif, Alireza Heiran

**Affiliations:** ^1^Student Research Committee, Shiraz University of Medical Sciences, Shiraz, Iran; ^2^Non-communicable Diseases Research Center, Shiraz University of Medical Sciences, Shiraz, Iran; ^3^Department of Epidemiology, School of Health, Research Center for Health Sciences, Shiraz University of Medical Sciences, Shiraz, Iran; ^4^Non-communicable Diseases Research Center, Department of Epidemiology, School of Health, Shiraz University of Medical Sciences, Shiraz, Iran

**Keywords:** delayed diagnosis, HIV, CD4 depletion model, Iran, AIDS

## Abstract

**Objective:**

Delayed diagnosis of HIV can lead to an inappropriate response to antiretroviral therapy (ART), rapid progression of the disease, and death. It can also carry harmful effects on public health due to the increment of transmission. This study aimed to estimate the duration of delayed diagnosis (DDD) in HIV patients in Iran.

**Methods:**

This hybrid cross-sectional cohort study was conducted on the national HIV surveillance system database (HSSD). Linear mixed effect models with random intercept, random slope, and both were used to estimate the parameters required for the CD4 depletion model to determine the best-fitted model for DDD, stratified by the route of transmission, gender, and age group.

**Results:**

The DDD was estimated in 11,373 patients including 4,762 (41.87%) injection drug users (IDUs), 512 (4.5%) men who had sexual contact with men (MSM), 3,762 (33.08%) patients with heterosexual contacts, and 2,337 (20.55%) patients who were infected through other routes of HIV transmission. The total mean DDD was 8.41 ± 5.97 years. The mean DDD was 7.24 ± 0.08 and 9.43 ± 6.83 years in male and female IDUs, respectively. In the heterosexual contact group, DDD was obtained as 8.60 ± 6.43 years in male patients and 9.49 ± 7.17 years in female patients. It was also estimated as 9.37 ± 7.30 years in the MSM group. Furthermore, patients infected through other transmission routes were found with a DDD of 7.90 ± 6.74 years for male patients and a DDD of 7.87 ± 5.87 years for female patients.

**Conclusion:**

A simple CD4 depletion model analysis is represented, which incorporates a pre-estimation step to determine the best-fitted linear mixed model for calculating the parameters required for the CD4 depletion model. Considering such a noticeably high HIV diagnostic delay, especially in older adults, MSM, and heterosexual contact groups, regular periodic screening is required to reduce the DDD.

## 1. Introduction

Human immunodeficiency virus (HIV) is one the leading causes of morbidity and mortality in developing countries. Globally, 38.4 million people were living with HIV in 2021; and ~650,000 people died from acquired immunodeficiency syndrome (AIDS)-related illnesses in 2021 (1). To end the HIV epidemic, United Nations Program on HIV and AIDS (UNAIDS) committed all countries to the 90-90-90 goals, in which 90% of patients should be aware of their condition, 90% received antiretroviral therapy, and 90% achieved viral suppression (2).

The prevalence of HIV/AIDS has been reported to be <1% in the general population. Iran has been ranked among the countries with a concentrated epidemic, as this infection is mainly concentrated among key individuals including people who inject drugs (PWIDs), men who have sex with men (MSM), and female sex workers (FSWs) (3, 4). That is, according to the latest available reports, the disease prevalence was 9.7–20.5% among injection drug users (IDUs) (5), 2.23% in prostitutes, 2.5% in prisoners, and 1.7% in homeless people (6–8). In addition, in a study on the HIV Case Registry System of Iran till late 2021, this is estimated that 53,000 people were living with HIV in Iran, of whom 43 and 30% were diagnosed and received antiretroviral therapy (ART), respectively, (5). Moreover, the United Nations Joint Program on HIV and AIDS (UNAIDS) estimates that more than 90,000 people in this country are living with HIV (3); however, the prevalence of the disease varies in high-risk groups.

People living with HIV/AIDS (PLWHA) who are unaware of their disease are responsible for 40% of the disease transmission in the United States. These individuals miss golden periods of improving their treatment outcomes and avoiding HIV transmission (9). However, with the increasing use of diagnostic tests, the share of HIV-infected people who are aware of their condition is steadily increasing, leading reduction in high-risk behaviors and an increase in the use of ART, which could eventually reduce the risk of transmission (9, 10). As a consequence, delayed diagnosis is mainly observed among migrants, high-risk groups, people with less access to medical screenings, those aged > 40 years, and those who have children (11).

Due to the variations in the definition of delayed diagnosis, the incidence of delayed diagnosis has been explicated differently. Studies conducted in the UK reported the incidence of 15 and 30% using the CD4 count < 200 and < 50 cells/μL definitions, respectively (10). In the United States, it is calculated as 43% using the CD4 count < 200 cells/μL definition (12), with a median delay of 3.0 years. In Italy, it is reported as 24% using the definition “diagnosis to AIDS occurrence interval of <8 weeks” (12). Moreover, in the Netherlands, a mean delay of 6.1 years was reported among MSM during 1984–1995, which declined to 2.6 years in 2011 (13).

Generally, most HIV/AIDS patients are not diagnosed at the early stage of the infection (14). There is often no history of regular HIV testing in newly diagnosed patients, leading to uncertainty about the duration of infection. Such information about the interval between the onset of infection to diagnosis would be essential for the health surveillance system to monitor HIV early detection strategies (i.e., motivation-enhancing strategies) as well as to determine the incidence and prevalence of the disease in a population (14). CD4 cell count depletion can be further applied to estimate other epidemic measures such as HIV incidence and prevalence. This model is based on information on biomarkers (e.g., CD4, HIV viral load) and demographic factors (e.g., age, sex), which can be further extended to include various determinants of health (15). Therefore, owing to the paucity of information on the duration of delayed diagnosis (DDD) in Iran, the present study sought to estimate the DDD among HIV patients.

## 2. Methods

### 2.1. Study population and database

This hybrid cross-sectional historical cohort study was conducted on the national HIV surveillance system database (HSSD) from 1993 to 2019. In Iran, all of the health centers affiliated with the medical universities, as well as the non-profit health centers, have to report and register new cases to receive routine HIV care according to the national protocol. The HSSD's mission was to act as the central hub for reporting, medical consultation, covering HIV screening and testing in high-risk groups, providing support services, and allocating routine HIV cares including periodic physician visits, CD4 cell count measurement, viral load testing, hepatitis C virus (HCV) and hepatitis B virus (HBV) testing, and other routine assessments. These measures were then registered in the embedded data management software in HSSD and sent to the counseling centers for behavioral disorders and the Iranian Center for Infectious Disease Control and Prevention (CDC). Since the HSSD was established on 8 December 1993, after excluding infants born with HIV/AIDS, 17,062 (mean age of 34.14 ± 10.77 years, 69.49% male patients) confirmed patients have been registered till 16 March 2019. The most prevalent high-risk groups were injection drug users (IDUs) (42.83%) and heterosexual contact subjects (33.83%). Furthermore, the mean baseline CD4 count was 404.92 ± 520.78 cells/μL. Details are available in Table 1. The study was performed according to the ethical guidelines expressed in the Declaration of Helsinki and the Strengthening of the reporting of observational studies in epidemiology (STORB) guideline.

**Table 1:** Characteristics of the national HIV/AIDS surveillance system database (HASS) from 8 December 1993 to 16 March 2019.

**Variable**	**Value**		
	**Total**	**Parameter estimation step**	**DDD estimation step**
	**(*****n*** = **17,062)**	**(*****n*** = **2,251)**	**(*****n*** = **11,373)**
**Sex**			
Male	11,856 (69.49)^a^	1,486 (66.01)	8,028 (70.58)
Female	5,206 (30.51)	765 (33.99)	3,345 (29.42)
Age, *yeas*	34.14 ± 10.77 [1–87]^b^	32.87 ± 10.24 [15–72]	34.56 ± 10.63 [7–82]
**IDUs**			
No	9,754 (57.17)	1,255 (55.76)	6,611 (58.13)
Yes	7,308 (42.83)	996 (44.24)	4,762 (41.87)
**Heterosexual contact**			
No	11,290 (66.17)	1,480 (65.75)	7,611 (66.92)
Yes	5,772 (33.83)	771 (34.25)	3,762 (33.08)
**MSM**			
No	16,231 (95.13)	2,153 (95.65)	10,861 (95.5)
Yes	831 (4.87)	98 (4.35)	512 (4.5)
**Vertical transmission**			
No	16,502 (96.72)	–^†^	–^†^
Yes	560 (3.28)		
**Other routes of transmission**			
No	14,098 (82.63)	1,865 (82.86)	9,036 (79.45)
Yes	2,964 (17.37)	386 (17.14)	2,337 (20.55)
Baseline CD4 count, cells/μL	404.92 ± 520.78 [20–11,830]	536.02 ± 415.60 [10–5,052]	436.15 ± 416.55 [12–4,502]

a Count (%).

b Mean ± standard deviation [minimum–maximum].

† Excluded from the study.

IDUs, injection drug users; MSM, male-to-male sexual contact.

### 2.2. Estimation of the duration of delayed diagnosis

The CD4 depletion model proposed by Song et al. (16) was utilized to estimate the DDD. The CD4 depletion model is a back-calculation method, which is widely used to calculate the incidence and prevalence of HIV. This method requires reporting system data, which is easier to be obtained than mathematical methods. This method is developed based on the assumption that the distribution of delayed diagnosis is constant over time. Based on this model (16), the duration of infection (DDD)—from initial infection to the HIV diagnosis or first CD4 cell count measurement—can be calculated using the following formula: $T_{i}=\frac{\left(\sqrt{{CD4}_{baseline}}-a_{i}\right)}{b_{i}}$, where T_i_ is the duration of infection (DDD), a_i_ is the random intercept, and b_i_ is the random slope.

To estimate the model parameters (a_i_ and b_i_), repeated CD4 cell count measurements were required before the initiation of ART. Therefore, at this step, only the patients who had repeated—at least two repeated—measurements of CD4 cell count before initiating ART were selected (historical cohort step). At this step, a total of 2,251 HIV patients (mean age of 32.87 ± 10.24 years, 66.01% male patients) were selected. The most prevalent high-risk groups were IDUs (44.24%) and heterosexual contact subjects (34.25%). In addition, the mean baseline CD4 count was 536.02 ± 415.60 cells/μL (Table 1).

Then, we used linear mixed models to estimate the model parameters, given that each patient was identified with different CD4 cell counts at the time of diagnosis. Furthermore, these models were required to predict the trend of CD4 cell counts for each patient over time. To select the best-fitted model, a linear mixed model with a random intercept, a linear mixed model with a random slope, and a linear mixed model with a random slope and intercept were assessed with the consideration of route of transmission, gender, and age group. Then, each model's output indices of diagnostic models comprising the Bayesian Information Criterion (BIC), Akaike Information Criterion (AIC), and log-likelihood were compared by likelihood ratio test (LRT). When two models fit significantly, the model with a narrower 95% confidence interval was selected as the final model (Supplementary material).

Following the estimation of the model parameters, those were generalized to all of the patients who had at least one pre-treatment (baseline) CD4 cell count measurement to estimate T_i_, considering the route of transmission, gender, and age group (cross-sectional step). At this step, a total of 11,373 HIV patients (mean age of 34.56 ± 10.63 years, 70.58% male patients) were selected. The most prevalent high-risk groups were IDUs (41.87%) and heterosexual contact subjects (33.08%). Furthermore, the mean baseline CD4 count was 436.15 ± 416.55 cells/μL (Table 1).

Then, to calculate the age at the onset of infection, the duration of infection (T_i_, DDD) was subtracted from the age at HIV diagnosis (or the first CD4 cell count measurement): *Age at onset of infection* = *Age at the first CD*4 *measurement* (*diagnosis*)− *T*_*i*_.

Data analysis was carried out using R programming software (version R 4.1.1, lme4 package) and Stata (version 15). Mean and standard deviation (SD), as well as median [interquartile range (IQR)] and 95% confidence interval (CI), were used to show the quantitative variables, and frequency and percentage were used to show the qualitative variables. Statistical tests, including *t*-test, one-way ANOVA, and Kaplan–Meier plot, were used to compare the DDD and age at the onset of infection according to transmission route, gender, and age group. The Kaplan–Meier survival analysis used DDD as the function of survival based on the four major transmission routes. In all analyses, a cut-off value of 0.05 was considered statistically significant for the P statistic.

## 3. Results

The calculated CD4 depletion model parameters (a_i_ and b_i_) are shown in Table 2. Furthermore, the linear mixed model with a random slope and intercept was selected as the best-fitted model among male IDUs and male patients with heterosexual contact(s), as well as the patients who were infected through other routes of transmission, while the linear mixed model with a random slope was selected for female patients of these groups, as well as MSM patients (Supplementary material).

**Table 2:** Estimation of CD4 depletion model parameters using the national HIV/AIDS surveillance system database (HASS) in male and female patients.

**Transmission category**	**Intercept (ai)**		**Slope (bi)**		
	**Value**	**SE**	**Coefficient**	**SE**	**95% CI**
**Males**					
**IDUs**					
15–25 y	24.18	0.7	−1.87	0.33	−2.56, −1.27
26–35 y	22.4	0.37	−1.26	0.14	−1.58, −0.94
>36 y	19.75	0.52	−0.64	0.19	−1.05, −0.26
**Heterosexual contact**					
≤ 32 y	22.74	0.46	−0.89	0.16	−1.27, −0.54
>32 y	20.13	0.55	−0.74	0.2	−1.17, −0.33
MSM^†^	20.8	0.73	−0.57	0.17	−0.92, −0.22
Others^†^	20.84	0.48	−0.88	0.17	−1.27, −0.51
**Females**					
IDUs^†(>16*y*)^	24.05	0.71	−2.06	0.9	−3.84, −0.28
**Heterosexual contact**					
≤ 32 y	24.09	1.04	−0.93	0.44	−1.80, −0.07
>32 y	21.32	0.99	−0.54	0.25	−1.05, −0.04

All parameters refer to square root transformed CD4 counts.

Slopes reflect changes per each year.

^†^All ages; small sample size in this particular group was not allowed for stratification.

SE, standard error; CI, confidence interval; IDUs, injection drug users; MSM, male-to-male sexual contact.

The estimated DDD and age at the onset of infection stratified by the transmission route, age, and gender are yielded in Table 3. The total mean DDD was 8.41 ± 5.97 [median (IQR): 6.92 (4.63, 13.10)] years. Accordingly, there was no significant difference between male and female patients with respect to the total mean DDD regardless of transmission route (8.52 ± 6.06 vs. 8.44 ± 6 years, *P* = 0.631). Mean DDD was 7.24 ± 0.08 and 9.43 ± 6.83 years in male and female IDUs, respectively. In the heterosexual contact group, it was 8.60 ± 6.43 years in male patients and 9.49 ± 7.17 years in female patients. In both IDUs and heterosexual contact groups, mean DDD was statistically higher in female patients than male patients (*P* < 0.001 in both). The mean DDD was 9.37 ± 7.30 years in the MSM group. Moreover, in patients who were infected through other routes of transmission, DDD was 7.90 ± 6.74 years in male patients and 7.87 ± 5.87 years in female patients, without no significant difference (*P* = 0.22).

**Table 3:** Duration of delayed diagnosis and age at the onset of infection in HIV patients using CD4 depletion model on the national HIV/AIDS surveillance system database (HASS).

**Group**	**Duration of delayed diagnosis (T**_**i**_ **)**		***P* ^†^**	**Age at the onset of infection**		***P* ^†^**
	**Male**	**Female**		**Male**	**Female**	
Total	8.52 ± 6.06	8.44 ± 6	0.631	28.44 ± 9.89	26.97 ± 11.02	<0.001
**IDUs**						
15–25	3.94 ± 2.84	–	–	23.21 ± 0.19	–	–
26–36	5.59 ± 4.11	–	–	27.75 ± 5.84	–	–
>36	10.11 ± 7.03	–	–	33.45 ± 9.52	–	–
*P* ^‡^	<0.001	–	–	<0.001	–	–
Total	7.24 ± 0.08	9.43 ± 6.83	<0.001	29.60 ± 0.11	26.71 ± 11.07	<0.001
**Heterosexual contact**						
15–25	7.31 ± 5.79	7.73 ± 6.28	0.507	15.41 ± 5.96	14.14 ± 6.19	0.122
26–36	8.34 ± 6.27	9.08 ± 6.60	0.042	22.85 ± 6.81	21.99 ± 6.73	0.028
>36	9.4 ± 6.75	11.16 ± 8.21	<0.001	35.30 ± 10.20	32.83 ± 10.95	0.001
*P* ^‡^	<0.001	<0.001	–	<0.001	<0.001	–
Total	8.60 ± 6.43	9.49 ± 7.17	<0.001	26.48 ± 10.69	24.05 ± 10.37	<0.001
**MSM**						
15–25	6.68 ± 5.71	–	–	17.21 ± 5.69	–	–
26–36	9.60 ± 7.16	–	–	22.28 ± 7.47	–	–
>36	10.15 ± 7.97	–	–	32.75 ± 1,041	–	–
*P* ^‡^	<0.001	–	–	<0.001	–	–
Total	9.37 ± 7.30	–	–	24.53 ± 9.99	–	–
**Others**						
15–25	7.08 ± 4.98	6.98 ± 5.74	0.883	18.02 ± 8.05	15.25 ± 7.43	0.007
26–36	7.49 ± 7.25	7.84 ± 60	0.538	25.22 ± 8.47	23.71 ± 6.72	0.019
>36	8.44 ± 6.55	8.17 ± 5.80	0.563	37.70 ± 10.91	36.83 ± 9.91	0.253
*P* ^‡^	0.002	0.272	–	<0.001	<0.001	–
Total	7.90 ± 6.74	7.86 ± 5.87	0.22	30.38 ± 12.06	28.54 ± 11.85	<0.001

† *P* derived from *t*-test for comparison of means of duration of delayed diagnosis and age at the onset of infection in male and female patients.

‡ *P* derived from the one-way ANOVA comparison of means of duration of delayed diagnosis and age at the onset of infection in different age groups.

IDUs, injection drug users; MSM, male-to-male sexual contact.

In all groups, older male patients had a significantly higher mean DDD than the younger patients [*P* (IDUs) < 0.001, *P* (heterosexual contact) < 0.001, *P* (MSM) < 0.001, *P* (other routes of transmission) = 0.002]. Furthermore, the mean DDD was significantly increased with age among female patients in the heterosexual contact group (*P* < 0.001). However, there was no statistical difference in mean DDD between the age groups of female patients who were infected through other routes of transmission (*P* = 0.272) (Table 3).

The total mean age at the onset of infection was 28.26 ± 10.04 [median (IQR): 27.77 (21.85, 33.83)] years. It was statistically higher in male patients compared to female patients (28.44 ± 9.89 vs. 26.97 ± 11.02 years, *P* < 0.001). The mean age at the onset of infection was 29.60 ± 0.11 and 26.71 ± 11.07 years in male and female IDUs, respectively, representing that male patients were infected at older ages (*P* < 0.001). Similarly, the mean age at the onset of infection in male patients who were infected through heterosexual contact was higher than that of female patients (26.48 ± 10.69 vs. 24.05 ± 10.37 years, *P* < 0.001). This measure was 24.53 ± 9.99 years in MSM. Furthermore, in patients who were infected through other routes of transmission, the mean age at the onset of infection was 30.38 ± 12.06 years in male patients and 28.54 ± 11.85 years in female patients, which was significantly higher in male patients (*P* < 0.001) (Table 3).

**Figure 1:**
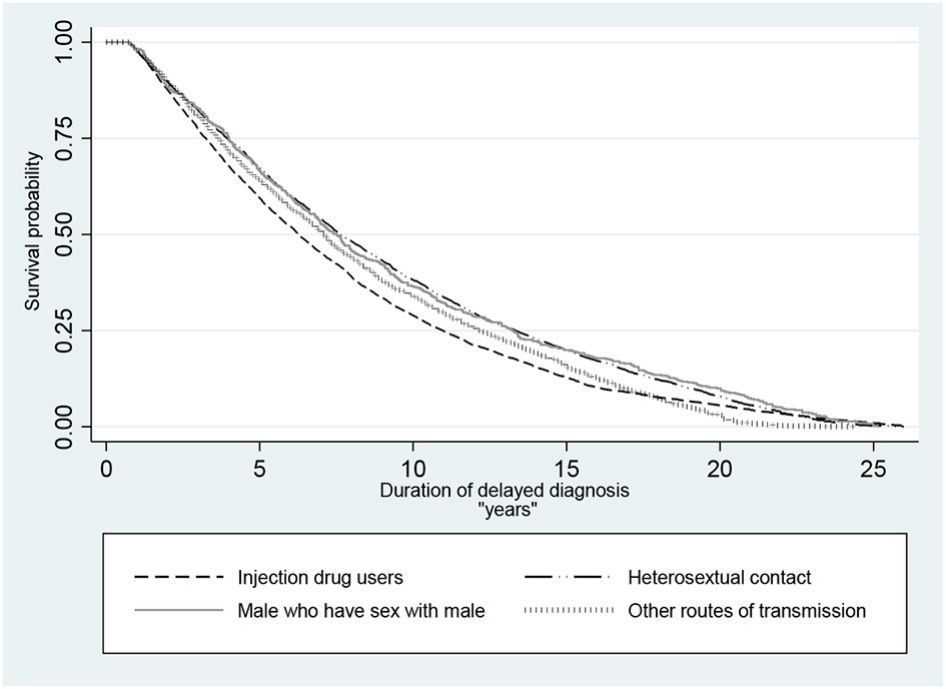
Kaplan–Meier survival probability of delayed diagnosis in HIV patients in Iran (*P* < 0.001 of log-rank test for equality of survivor functions).

Using survival analysis, the median DDD was 6.27 (95% CI: 6.10, 6.47) years in the IDUs group, 7.61 (95% CI: 7.38, 7.93) years in the heterosexual contact group, 7.50 (95% CI: 6.87, 7.99) years in the MSM group, and 7.05 (95% CI: 6.82, 7.31) years in patients who infected through other routes of transmission. The difference among these groups was statistically significant (*P* < 0.001) (Figure 1).

## 4. Discussion

In the present study, the DDD and age at the initial HIV infection were estimated using the data from the national HIV/AIDS surveillance system in Iran. The results majorly showed that the mean DDD was widely different based on the transmission route. Accordingly, the shortest and longest DDD were observed in IDUs and MSM groups, respectively. In addition, generally, the mean DDD was significantly prolonged with an increase in age in all the transmission routes. In addition, female patients had a longer DDD compared to male patients.

Until now, a few studies have estimated the duration from HIV infection to its diagnosis, most of which were qualitatively carried out with varying definitions of delayed diagnosis; i.e., time-based definition (less than a year from diagnosis to the development of AIDS), CD4 count at the time of diagnosis (ranged 50–350 μl), or clinically symptomatic at the time of diagnosis (10). Therefore, these studies reported different duration of HIV delayed diagnosis estimates, making it sophisticated to compare them and draw a big picture (10).

In the current study, the mean and median DDD were 8.41 and 6.92 years, respectively. Similarly, Hall et al. (17) used data from the National HIV Surveillance System (NHSS) in the United States to estimate DDD during 2014–2018 based on a CD4 depletion model analysis. They found that the median DDD decreased from 3.58 years in 2014 to 3.33 years in 2018, indicating a 1.5% annual decrease. Another study using the CD4 depletion model in the United States in 2013 reported the mean and median of DDD as 7 and 5.4 years, respectively. In 2011 also, the mean and median DDD were estimated at 5.6 and 3.6 years, respectively (17). Furthermore, in van Sighem et al.s' study using multi-state analysis of HIV surveillance data of the Netherlands, the mean DDD was 6.1 years in MSM patients during 1984–1995, which showed a decrease to 2.6 years in 2011 (13). In addition to the existing definition heterogeneity, these differences might be attributed to the diverse strategies of the HIV surveillance systems including screening programs in high-risk groups, availability of diagnostic tests, periodic HIV care, stigma and discrimination, socioeconomic status, and access to hidden high-risk groups in the society.

In line with the present study, the previous studies demonstrated that IDUs had a shorter DDD in comparison to those with other routes of transmission (18, 19). Similarly, Lott et al. (20), mentioned that the stigma and discrimination of HIV in society, especially in sensitive groups such as MSM and FSW, can cause more difficult access and fewer tests; therefore, delays in diagnosis can be reported more in such groups. In addition, it might be also partially attributed to the higher rate of screening tests in this high-risk population.

Most previous studies showed that older age—especially > 40 years—generally, is a risk factor for the delayed diagnosis of HIV (10, 21, 22). In a study conducted in Iran, using the definition “interval of <3 months for diagnosis to CD4 cell count of <350”, they found a prevalence of 75.3% late diagnosis.

The results also indicated that age and comorbid infection with HCV increase the odds of late diagnosis by 72 and 65%, respectively (23). In another study conducted by Mohammadi et al. (24), on data from the Iranian population, the prevalence was reported as 58.2%, according to the same definition as the just-mentioned study. Furthermore, the authors showed that delayed diagnosis was 3.5 times higher in patients aged > 50 years, 2.89 times higher in patients with blood-transmitted HIV, 2.06 times in patients with comorbid tuberculosis infection, and 1.38 times higher in male patients. In the present study, however, the prevalence of delayed diagnosis was lower in male patients than in female patients, which could be related to the study design and use of different definitions.

Another finding of our study was the higher mean DDD in female patients, which was in line with the results of the study carried out by Mugavero et al. and Sabin et al. (21, 25). A study conducted by Adler et al. (10), which considered CD4 < 50 as the indicator of delayed diagnosis, showed that female patients more often presented late. Furthermore, it is shown that inpatient diagnosis of HIV infection is significantly more common among women and older patients (21). However, a majority of the literature is against this finding (10, 26, 27). Of note, such differences might result from cultural differences as well as a social stigma among Iranian girls and women. Differences in study design could be also influential.

In addition, DDD was highest among the MSM group followed by the heterosexual contact group and patients who were infected through other routes of transmission. A European study also demonstrated that MSM had the highest prevalence of delayed diagnosis (10). However, again, an ample deal of evidence is against this finding, reporting the highest prevalence of delayed diagnosis in the heterosexual contact group (17, 28, 29). In another study in Spain, the risk of delayed diagnosis was higher in patients with a history of heterosexual contact and IDUs compared to the MSM group (30). In Iran, MSM and heterosexual contact groups have apparently the highest rate of delayed diagnosis, probably due to cultural and social issues. In a study conducted in Iran, nearly all patients reported experiencing stigma and discrimination by their healthcare providers in various contexts. From the perspective of patients, healthcare providers' fear of becoming infected with HIV, and religious and negative opinions about HIV, lead to high levels of stigma (31). Moreover, they are usually considered as a hidden and neglected group of the community who usually do not declare their sexual contact experiences and have not a sufficient access to HIV testing and care compared to other people.

Learned from the literature, common risk factors associated with delayed diagnosis of HIV include migration, older age, heterosexuality, male gender, living in less HIV-prevalent areas, and having a child (11). Despite the availability of free diagnostic tests and consultation services and the guaranteed confidentiality of patients' identity data in harm reduction centers in Iran, the relatively low rate of HIV screening tests might still remain an obstacle to the higher delayed diagnosis rates in this country. This issue can be attributed to unawareness about the locations of testing and harm reduction centers, illiteracy about the disease, unwillingness and resistance to undergo screening tests, and fear of positive testing and disease-related stigma (32). By and large, it is necessary to conduct more studies about the reasons for lower rates of HIV testing among different groups in Iran.

Delayed HIV diagnosis can negatively affect target HIV outcomes including the rapid progression of the disease, death, inappropriate response to ART, reduced general health due to the possibility of transmission, and socioeconomic consequences (33, 34). Therefore, to achieve the UNAIDS “90-90-90” targets (35), effective programs for periodic screening among high-risk and hidden groups are demanded.

### 4.1. Limitations

To calculate the parameters of the CD4 depletion model, repeated measurements of CD4 cell count before the initiation of treatment were required, which inevitably decreased the size of the population in the first step of the analysis. The limited number of patients in the MSM group was another limitation of the study, which could affect the accuracy of the parameters' estimation. Furthermore, because of the small sizes of groups with transfusion-transmitted and occupational HIV infection, as well as those with an unknown route of transmission, we aggregated them into the “Other routes of transmission” group; therefore, this study was mute on DDD estimations in these groups of HIV/AIDS patients.

### 4.2. Strengths of the study

This study was the first investigation in Iran, carried out to estimate the HIV/AIDS DDD in a large sample size within a 27-year time frame. Up to now, different definitions of delayed diagnosis have been employed in various studies, making the comparison a complex work. Nonetheless, the present study estimated the DDD using the database of the national HIV/AIDS surveillance system by using the CD4 depletion model.

## 5. Conclusion

Delayed HIV diagnosis is important for controlling the disease and preventing its transmission in the community. In this study, we represented a simple CD4 depletion model analysis using the real-world surveillance system data to estimate DDD, which incorporates a pre-estimation step to determine the best-fitted linear mixed model for calculating the parameters required for the CD4 depletion model. We showed prolonged HIV diagnostic delays, especially in older, MSM, and heterosexual contact populations, as well as a shifted pattern of disease transmission in Iran. Hence, this is recommended to consider regular periodic screening and increase the frequency of diagnostic testing in these groups to reduce DDD.

## Data availability statement

The original contributions presented in the study are included in the article/Supplementary material, further inquiries can be directed to the corresponding author.

## Ethics statement

The studies involving human participants were reviewed and approved by the Research Ethics Committee of Shiraz University of Medical Sciences (IR.SUMS.SCHEANUT.REC.1400.045). Written informed consent to participate in this study was provided by the participants' legal guardian/next of kin. Written informed consent was not obtained from the individual(s), nor the minor(s)' legal guardian/next of kin, for the publication of any potentially identifiable images or data included in this article.

## Author contributions

MSh: final analysis, providing the main idea of the study, and methodology. AM: final analysis, developing the idea, and revising the final manuscript. JH: developing the idea and revising the final manuscript. MSe: data analysis. AH: revising the final manuscript. All authors approved the final version of the manuscript.
