# Supplementary material for: Duration of delayed diagnosis in HIV/AIDS patients in Iran: a CD4 depletion model analysis
Source: Front Public Health. 2023 Apr 17;11:1029608. doi: 10.3389/fpubh.2023.1029608 (PMC10149797; doi:10.3389/fpubh.2023.1029608)
Supplement: Supplementary file 1 [file Data_Sheet_1.PDF]

Supplementary files

1- Model selection for the estimation of CD4 depletion model parameters in injection drugs users

1-1- Model selection: Comparison of the linear mixed effect models (random intercept, random slope, and random intercept and slop) in male injection drug users aged 15-25 years

Model	N parameters	AIC	BIC	Log-likelihood	P *
Random intercept	4	2612.5	2628.4	-1302.2	
Random slop	6	2588.6	2612.6	-1288.3	0.556
Random intercept and slop	5	2587.0	2606.9	-1288.5	<0.001
*P for the likelihood ratio test					

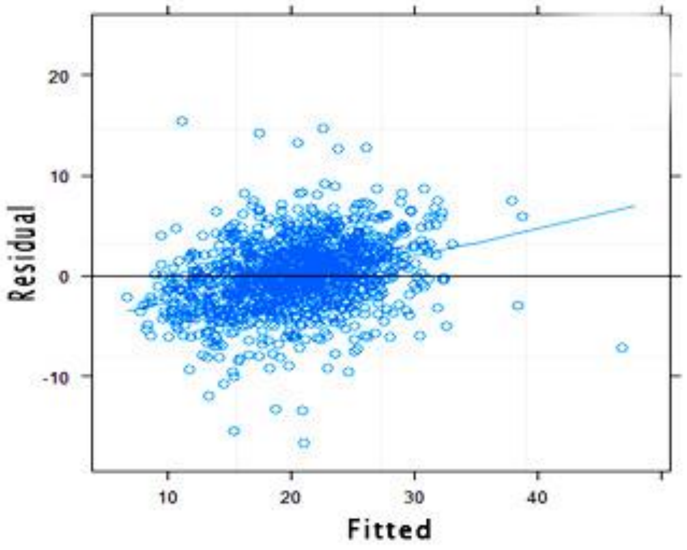

Plot 1. The residuals vs. fitted values plot in random intercept and slop model amongst male injection drug users aged 15-25 years

1-2- Model selection: Comparison of the linear mixed effect models (random intercept, random slope, and random intercept and slope) in male injection drug users aged 26-35 years

Model	N parameters	AIC	BIC	Log likelihood	P *
Random intercept	4	8380.7	8401.4	-4186.3	
Random slop	6	8378.0	8409.1	-4183.0	0.282
Random intercept and slop	5	8377.2	8403.1	-4183.6	0.019

* P for the likelihood ratio test

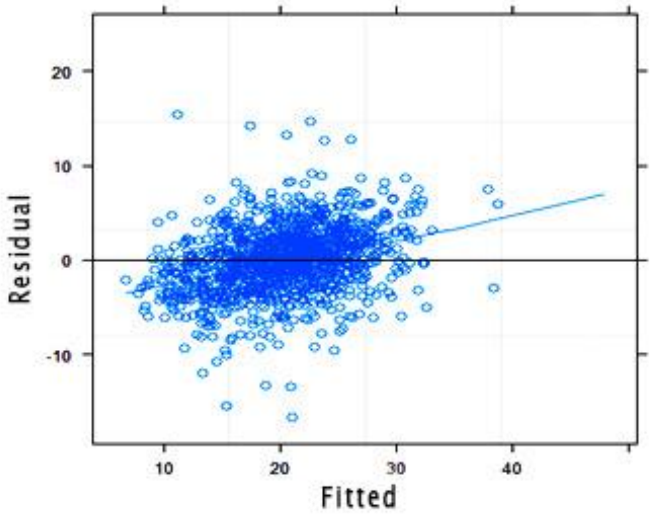

Plot 2. The residuals vs. fitted values plot in random intercept and slop model amongst male injection drug users aged 26-35 years

1-3- Model selection: Comparison of the linear mixed effect models (random intercept, random slope, and random intercept and slope) in male injection drug users aged >36 years

Model	N parameters	AIC	BIC	Log likelihood	P *
Random intercept	4	5750.7	5769.8	-2871.3	
Random slop	6	5736.0	5764.7	-2862.0	0.049
Random intercept and slop	5	5737.9	5761.8	-2863.9	<0.001
* P for the likelihood ratio test					

Comparison of two models by confidence intervals for the parameters in male injection drug users aged >36 years

Model	Intercept, 95% (CI)	Difference	Slop 95% (CI)	Difference
Random slop	18.72, 20.85	2.3	-1.09, -0.24	0.85
Random intercept and slop	18.76, 20.75	1.99	-1.05, -0.26	0.79

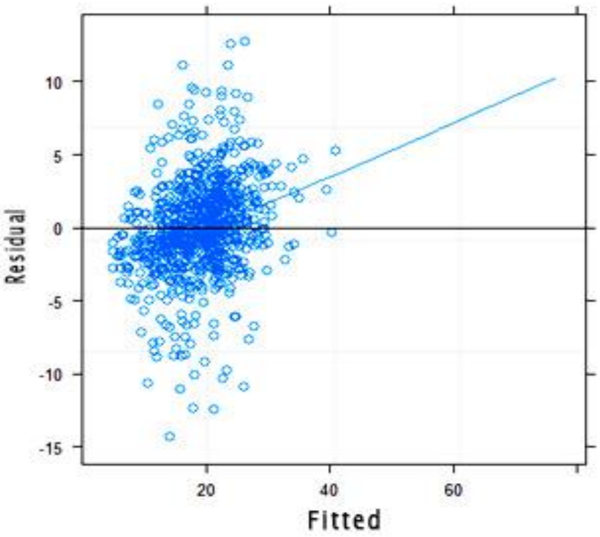

Plot 3. The residuals vs. fitted values plot in random intercept and slop model amongst male injection drug users aged >36 years

1-4- Model selection: Comparison of the linear mixed effect models (random intercept, random slope, and random intercept and slope) in female injection drug users

Model	N parameters	AIC	BIC	Log-likelihood	P *
Random intercept	4	645.69	656.19	-318.84	
Random slop	6	645.78	661.53	-316.89	0.058
Random intercept and slop	5	647.35	660.48	-318.68	0.564
* P for the likelihood ratio test					

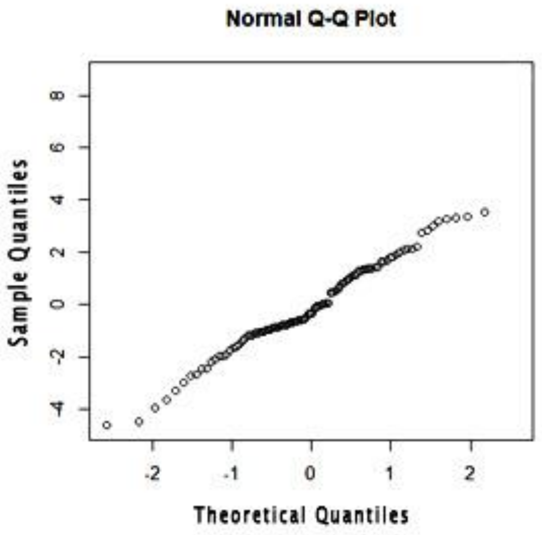

Plot 4. QQ plot for residuals for random slop model amongst female injection drug users

2- Model selection for the estimation of CD4 depletion model parameters in heterosexual contact

2-1- Model selection: Comparison of the linear mixed effect model (random intercept, random slope, and random intercept and slope) in heterosexual males aged <32 years

Model	N parameters	AIC	BIC	Log-likelihood	P *
Random intercept	4	6556	6575.7	-3274	
Random slop	6	6553.2	6582.7	-3270.6	0.098
Random intercept and slop	5	6554	6578.5	-3272.	0.043
* P for the likelihood ratio test					

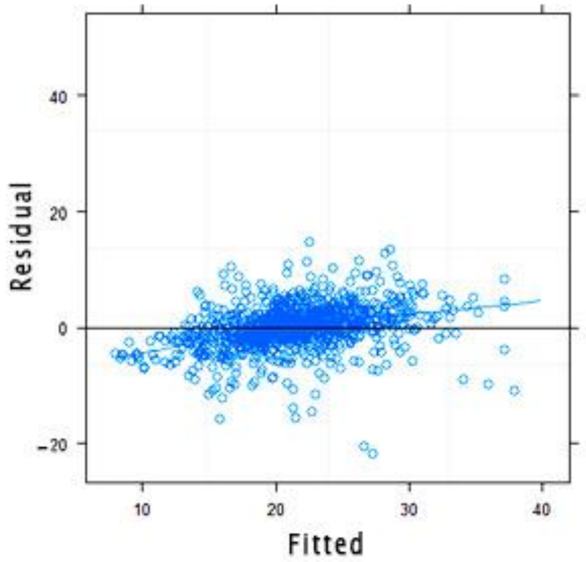

Plot 5. The residuals vs. fitted values plot in random intercept and slop model amongst heterosexual males aged <32 years

2-2- Model selection: Comparison of the linear mixed effect models (random intercept, random slope, and random intercept and slope) in heterosexual males >32 years

Model	N parameters	AIC	BIC	Log- likelihood	P *
Random intercept	4	4933.8	4952.4	-2462.9	
Random slop	6	4924.1	4952	-2456.1	0.10
Random intercept and slop	5	4924.7	4947.9	-2457.3	<0.001
* P for the likelihood ratio test					

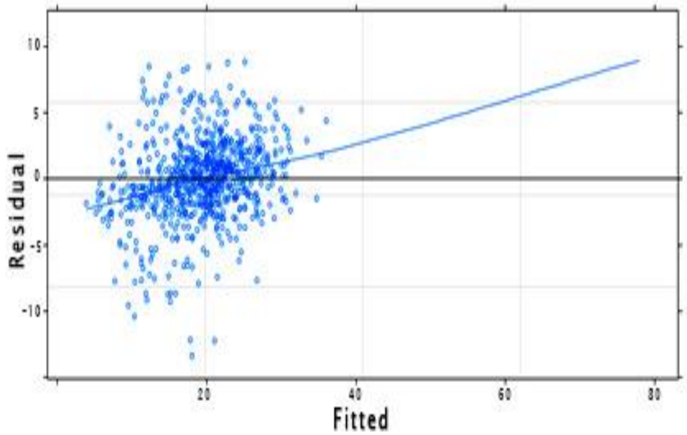

Plot 6. The residuals vs. fitted values plot in random intercept and slop model amongst heterosexual males >32 years

2-3- Model selection: Comparison of the linear mixed effect models (random intercept, random slope, and random intercept and slop) in heterosexual females aged ≤32 years

Model	N parameters	AIC	BIC	Log-likelihood	P *
Random intercept	4	1473.1	1486.9	-732.57	
Random slop	6	1470.4	1491.1	-729.20	0.016
Random intercept and slop	5	1474.1	1491.3	-732.07	0.316
* P for the likelihood ratio test					

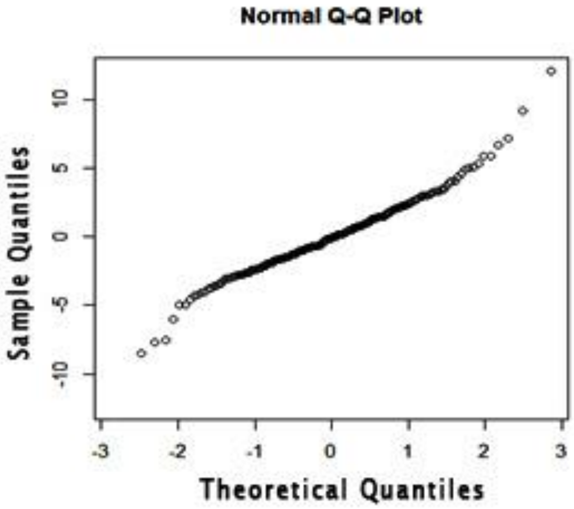

Plot 7. QQ plot for residuals for random slop model amongst heterosexual females aged ≤32 years

2-4- Model selection: Comparison of the linear mixed effect models (random intercept, random slope, and random intercept and slop) in heterosexual females aged >32 years

Model	N parameters	AIC	BIC	Log-likelihood	P *
Random intercept	4	933	945.05	-462.50	
Random slop	6	936	954.80	-462.37	0.60
Random intercept and slop	5	935	950.06	-462.50	1
* P for the likelihood ratio test					

Comparison of two models by confidence intervals for the parameters in heterosexual females aged >32 years

Model	Intercept, 95% (CI)	Difference	Slop 95% (CI)	Difference
Random slop	19.22, 23.30	4.08	-1.05, -0.040	1.09
Random intercept and slop	19.15, 23.21	4.06	-1.30, 0.034	1.33

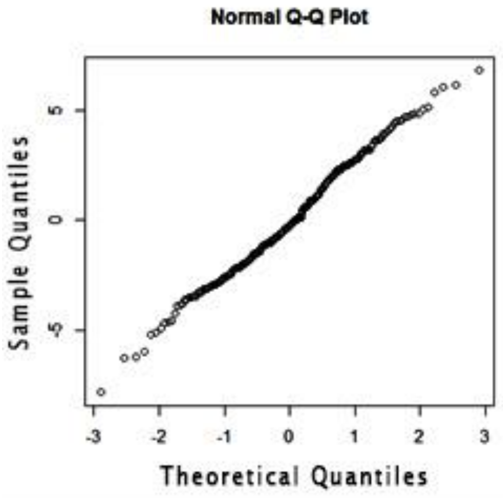

Plot 8. QQ plot for residuals for random slop model amongst heterosexual females aged >32 years

3- Model selection for the estimation of CD4 depletion model parameters in male-to-male sexual contact (MSM)

3-1- Model selection: Comparison of the linear mixed effect models (random intercept, random slope, and random intercept and slope) in MSM

Model	N parameters	AIC	BIC	Log-likelihood	P *
Random intercept	4	1616.3	1630.7	-804.16	
Random slop	6	1620.2	1641.8	-804.10	0.73
Random intercept and slop	5	1618.3	1636.3	-804.16	1
* P for the likelihood ratio test					

Comparison of two models by confidence intervals for the parameters in MSM

Model	Intercept, 95% (CI)	Difference	Slop 95% (CI)	Difference
Random slop	21.29, 24.26	2.97	-1.01, -0.16	0.85
Random intercept and slop	21.44, 24.41	2.97	-1.18, -0.25	0.9

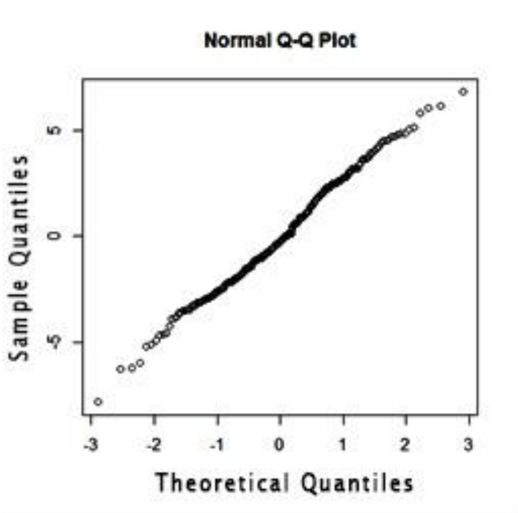

Plot 9. QQ plot for residuals for random slop model amongst MSM

4- Model selection for the estimation of CD4 depletion model parameters in other transmission category

4-1- Model selection: Comparison of the linear mixed effect models (random intercept, random slope, and random intercept and slop) in patients with other modes of transmission

Model	N parameters	AIC	BIC	Log-likelihood	P *
Random intercept	4	6410.6	6430.1	-3201.3	
Random slop	6	6409.4	6438.8	-3198.7	0.159
Random intercept and slop	5	6409.4	6433.9	-3199.7	0.076
* P for the likelihood ratio test					

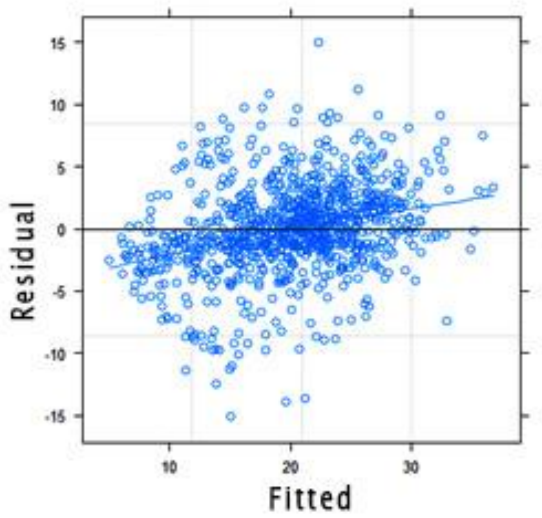

Plot 10. The residuals vs. fitted values plot in random intercept and slop model amongst patients with other modes of transmission
